# Supplementary material for: Jag1 insufficiency alters liver fibrosis via T cell and hepatocyte differentiation defects
Source: EMBO Mol Med. 2024 Oct 2;16(11):2946–75. doi: 10.1038/s44321-024-00145-8 (PMC11554675; doi:10.1038/s44321-024-00145-8)
Supplement: Supplementary file 1 — Appendix [file 44321_2024_145_MOESM1_ESM.pdf]

Jag1 Insufficiency Alters Liver Fibrosis via T Cell and Hepatocyte Differentiation Defects

Jan Mašek[1,2,3]*, Iva Filipovic[4☯], Noémi Van Hul[1☯], Lenka Belicová[1☯], Markéta Jiroušková[5#], Daniel V. Oliveira[2#], Anna Maria Frontino[2#], Simona Hankeova[1#], Jingyan He[1#], Fabio Turetti[2], Afshan Iqbal[1], Igor Červenka[1], Lenka Sarnová[5], Elisabeth Verboven[1], Tomáš Brabec[2], Niklas K. Björkström[4], Martin Gregor[5], Jan Dobeš[2], Emma R. Andersson[1,3]*

[☯, #] equal contribution

Corresponding authors' contact information: [*] correspondence to jan.masek@natur.cuni.cz and/or emma.andersson@ki.se

Affiliation:

[1] Department of Cell and Molecular Biology, Karolinska Institute, Stockholm, SE-171 77 Solna, Sweden.

[2] Department of Cell Biology, Faculty of Science, Charles University, Viničná 7, 128 00 Prague 2, Czech Republic.

[3] Department of Biosciences and Nutrition, Karolinska Institute, Huddinge 14183, Sweden

[4] Center for Infectious Medicine, Department of Medicine Huddinge, Karolinska Institutet, Karolinska University Hospital, Stockholm, Sweden

[5] Laboratory of Integrative Biology, Institute of Molecular Genetics of the Czech Academy of Sciences, Vídeňská 1083, Prague, Czech Republic.

Appendix Figures:

Appendix Figure S1, page 2

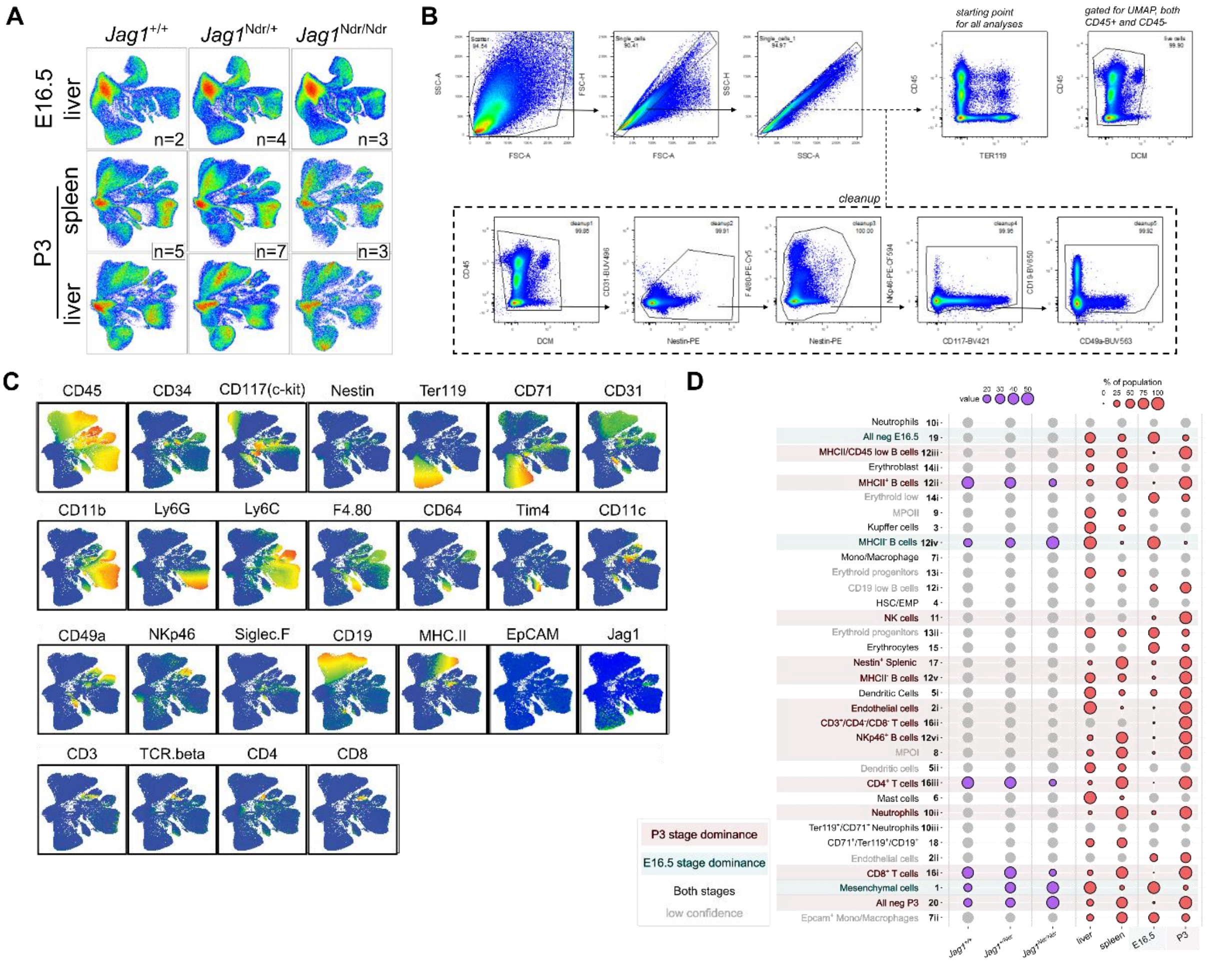

Appendix Figure S1: ***25-colour Flow cytometry profiling of embryonic and postnatal liver and spleen reveals enrichment of B cells and depletion of T- cell populations in Jag1$^{Ndr/Ndr}$ mice.*** (**A**) Genotype, stage, or organ contribution to the composition of the Flow cytometry spleen and/or liver samples from E16.5 and P3 *Jag1$^{+/+}$*, *Jag1$^{Ndr/+}$*, and *Jag1$^{Ndr/Ndr}$* mice. (**B**) Flow cytometry gating strategy. (**C**) UMAP projections of the cells expressing marker proteins across the aggregated 25-colour Flow cytometry dataset of 194,999 cells sampled from E16.5 and P3 livers, and P3 spleens of *Jag1$^{+/+}$*(n=7 biological replicates), *Jag1$^{Ndr/+}$*(n=11 biological replicates), and *Jag1$^{Ndr/Ndr}$* (n=6 biological replicates) mice (**D**) Dot plot depicting relative frequencies of the identified cell populations across genotypes, tissues, and developmental stages. MPO, myeloid progenitors; HSC, haematopoietic stem cells; EMP, erythroid-myeloid progenitor.
