# Supplementary material for: Jag1 insufficiency alters liver fibrosis via T cell and hepatocyte differentiation defects
Source: EMBO Mol Med. 2024 Oct 2;16(11):2946–75. doi: 10.1038/s44321-024-00145-8 (PMC11554675; doi:10.1038/s44321-024-00145-8)
Supplement: Supplementary file 3 — Expanded View Figures [file 44321_2024_145_MOESM3_ESM.pdf]

Expanded View Figures

Figure EV1. Single-cell profiling of the developing and postnatal liver reveals a cell population shift from embryonic erythropoiesis to postnatal immune surveillance in *Jag1*$^{+/+}$, *Jag1*$^{Ndr/+}$, and *Jag1*$^{Ndr/Ndr}$ mice.

(**A**) Violin plots of read counts (nCount_RNA), unique gene counts (nFeature_RNA), and percentage of mitochondrial mRNA (percent-mt) across the cell types identified in the *Jag1*$^{+/+}$, *Jag1*$^{Ndr/+}$, and *Jag1*$^{Ndr/Ndr}$ livers at E16.5 and P3 ($n = 2$ each). (**B**) Genotype, stage, or organ contribution to the composition of the scRNAseq dataset of E16.5 and P3 *Jag1*$^{+/+}$, *Jag1*$^{Ndr/+}$, and *Jag1*$^{Ndr/Ndr}$ mice. (**C**) Dot plot of the SCT-normalized mRNA marker expression of 78 DEGs characteristic for the individual cell types identified in (**B**). (**D**) Bar graph representing the relative proportion of parenchymal and Kupffer cells (clusters 1–6) versus non-parenchymal cells (clusters 7–20) in dissociated *Jag1*$^{+/+}$, *Jag1*$^{Ndr/+}$, and *Jag1*$^{Ndr/Ndr}$ livers at E16.5 and P3 ($n = 2$ each). Graph represents mean ± SD. (**E**) Graph depicting relative cell type contribution in dissociated *Jag1*$^{+/+}$, *Jag1*$^{Ndr/+}$, and *Jag1*$^{Ndr/Ndr}$ livers at E16.5 and P3 normalized to the total sample cell count.

 © The Author(s)

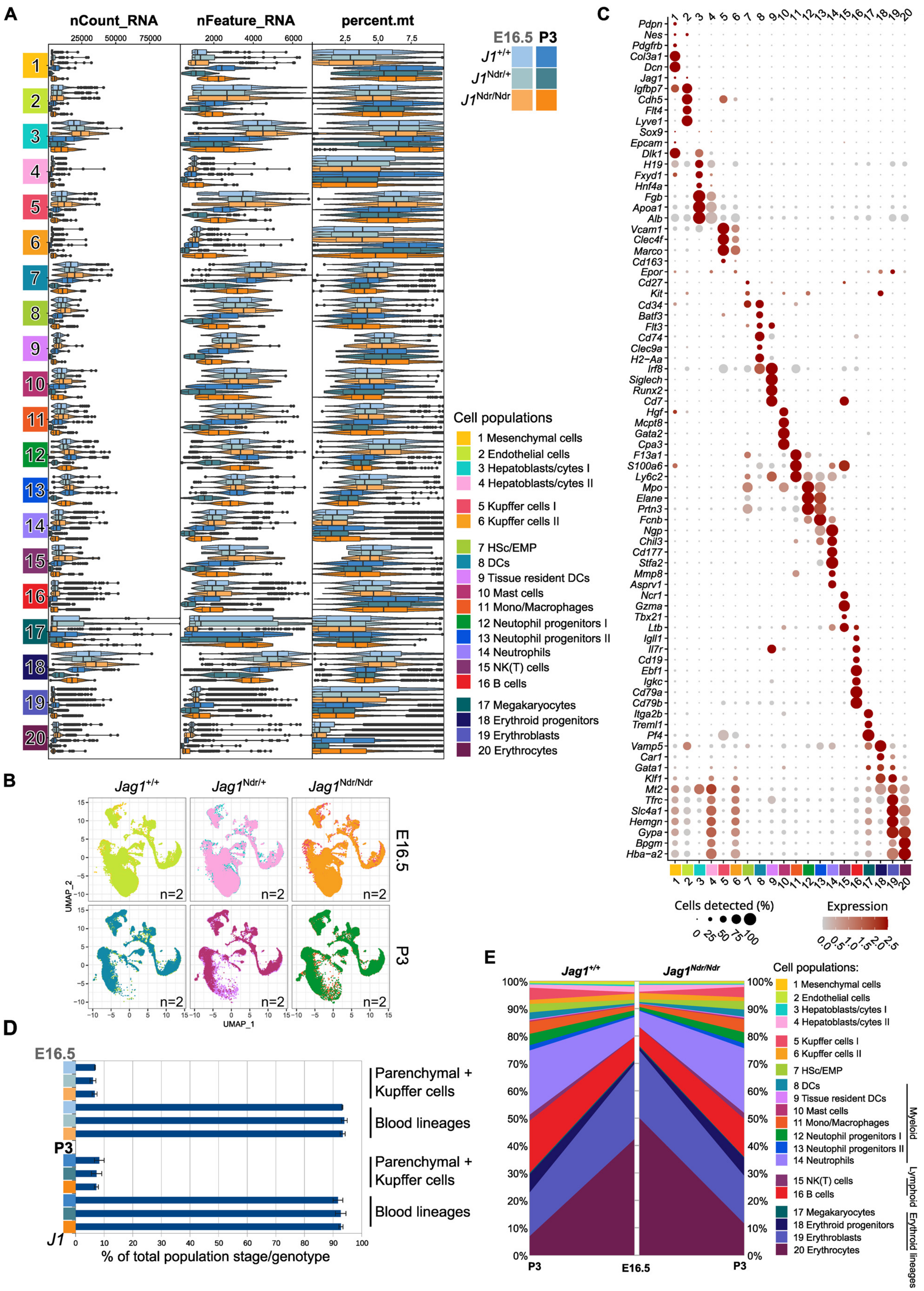
A
nCount_RNA
nFeature_RNA
percent.mt
E16.5 P3
J1+/+
J1Ndr/+
J1Ndr/Ndr
Cell populations
1 Mesenchymal cells
2 Endothelial cells
3 Hepatoblasts/cytes I
4 Hepatoblasts/cytes II
5 Kupffer cells I
6 Kupffer cells II
7 HSc/EMP
8 DCs
9 Tissue resident DCs
10 Mast cells
11 Mono/Macrophages
12 Neutophil progenitors I
13 Neutophil progenitors II
14 Neutrophils
15 NK(T) cells
16 B cells
17 Megakaryocytes
18 Erythroid progenitors
19 Erythroblasts
20 Erythrocytes
B
Jag1+/+
Jag1Ndr/+
Jag1Ndr/Ndr
E16.5
P3
UMAP_1
UMAP_2
n=2
C
Cells detected (%)
Expression
D
E16.5
P3
Parenchymal + Kupffer cells
Blood lineages
% of total population stage/genotype
E
Jag1+/+
Jag1Ndr/Ndr
P3
E16.5
Cell populations:
Myeloid
Lymphoid
Erythroid
Blood lineages

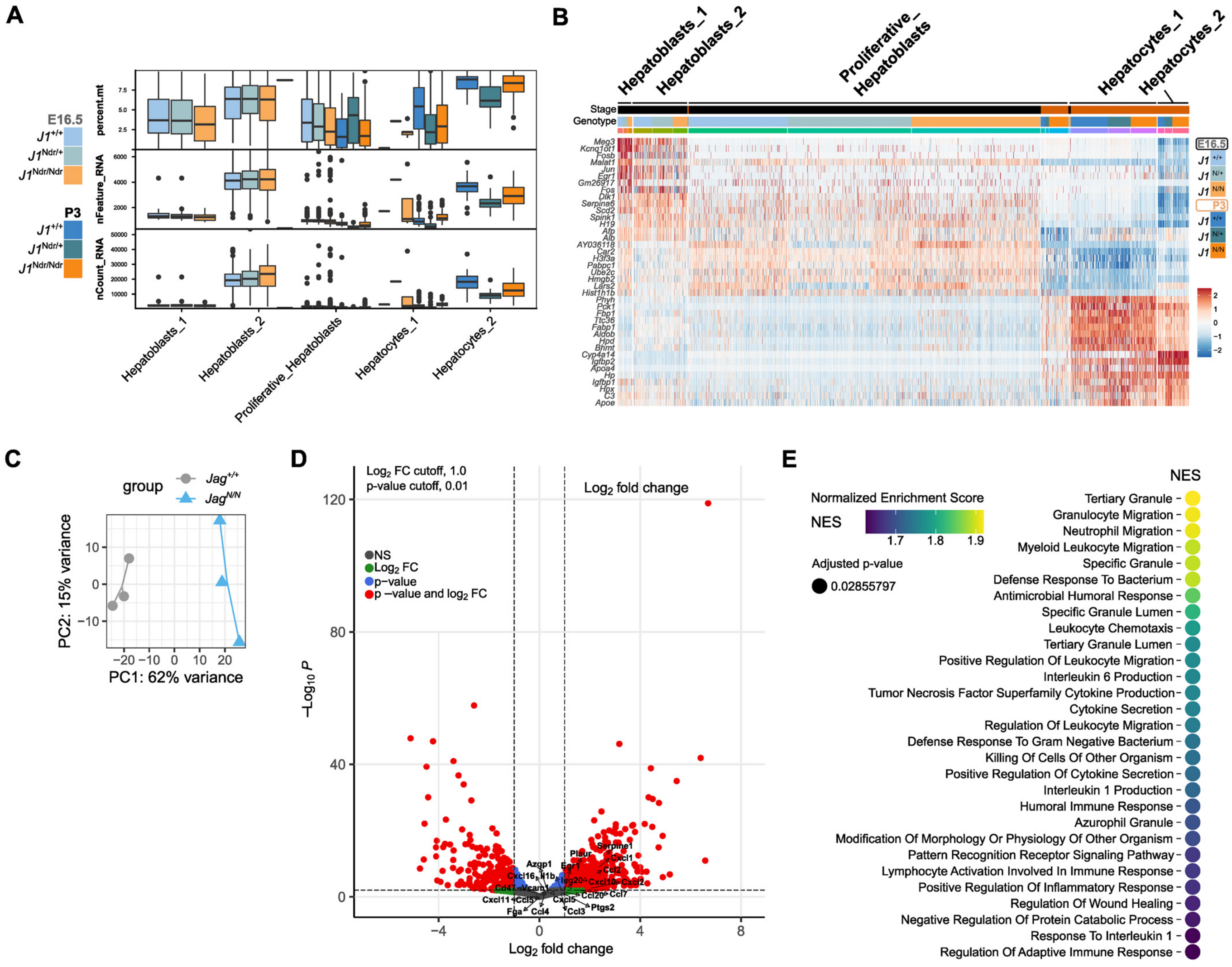

Figure EV2. Altered gene expression profile of hepatocytes and whole livers in *Jag1*$^{Ndr/Ndr}$ animals.

(**A**) Box and whiskers plot of the percentage mitochondrial mRNA (percent.mt), unique gene counts (nFeature_RNA), and read counts (nCount_RNA) across the hepatocyte-like cell types identified in the *Jag1*$^{+/+}$, *Jag1*$^{Ndr/+}$, and *Jag1*$^{Ndr/Ndr}$ livers by scRNA seq at E16.5 and P3 ($n = 2$ each). Center line indicates Median (50th percentile). The lower and upper bound of box indicate Q1, and Q3 (25th and 75th percentiles), respectively. Lower and upper whisker indicate min and max values within 1.5 * IQR below Q1 and above Q3, respectively. (**B**) Heatmap of the top 8 mRNA markers for each cluster. (**C**) PCA distribution of the bulk RNAseq samples from Andersson et al, 2018, re-analyzed in Fig. 2 and Fig. EV2. (**D**) Volcano plot of the DEGs of *Jag1*$^{Ndr/Ndr}$ vs. *Jag1*$^{+/+}$ liver at P10. Pro-inflammatory markers of activated hepatocytes are highlighted. Significance was confirmed via Benjamini–Hochberg *P* value correction. (**E**) Top29 GO pathways enriched in the *Jag1*$^{Ndr/Ndr}$ livers, NES (normalized enrichment score) indicates overlap of genes across the pathways. Significance was confirmed via false-discovery rate (FDR) statistic with 25% cutoff.

 © The Author(s)

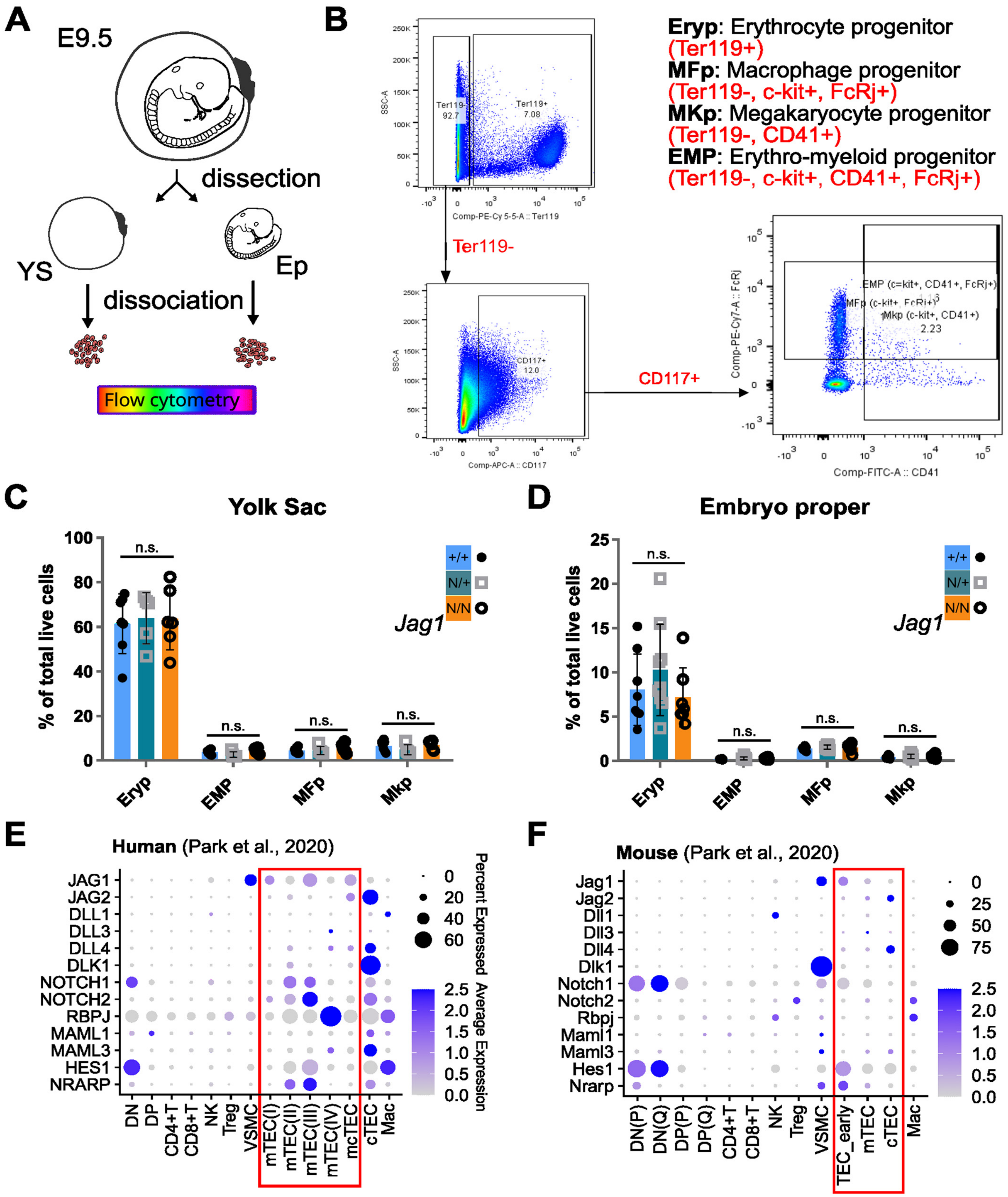
A
E9.5
dissection
YS
Ep
dissociation
Flow cytometry
B
Eryp: Erythrocyte progenitor (Ter119+)
MFp: Macrophage progenitor (Ter119-, c-kit+, FcRj+)
MKp: Megakaryocyte progenitor (Ter119-, CD41+)
EMP: Erythro-myeloid progenitor (Ter119-, c-kit+, CD41+, FcRj+)
Ter119-
CD117+
Comp-PE-Cy 5-5-A :: Ter119
Comp-APC-A :: CD117
Comp-FITC-A :: CD41
Comp-PE-Cy7-A :: FcRj
C
Yolk Sac
% of total live cells
Jag1
+/+
N/+
N/N
n.s.
Eryp
EMP
MFp
Mkp
D
Embryo proper
E
Human (Park et al., 2020)
Percent Expressed
Average Expression
F
Mouse (Park et al., 2020)

Figure EV3. The proportions of early embryonic hematopoietic progenitor populations are not altered in *Jag1*$^{Ndr/Ndr}$ embryo proper (EP) and yolk sac (YS).

(**A**) Schematic of the experiment. E9.5 embryos were harvested and yolk sac (YS) and embryo proper (EP) were processed separately for flow cytometry analysis. (**B**) Gating strategy for identification of the Erythrocyte progenitors (Eryp), Macrophage progenitors (MFp), Megakaryocyte progenitors (MKp), and Erythro-myeloid progenitors (EMP) after dead cell exclusion. (**C**, **D**) Relative proportion of live Eryp, EMP, MFp, and MKp cells from *Jag1*$^{Ndr/Ndr}$ ($n = 7$), *Jag1*$^{Ndr/+}$ ($n = 9$), and *Jag1*$^{+/+}$ ($n = 8$) yolk sac (**C**) and whole E9.5 embryos (**D**). Graph represents mean ± SD. (**E**, **F**) Dot plot of the re-analyzed median scaled ln-normalized mRNA expression of Notch signaling components in human (**E**) and mouse (**F**′) thymic cell populations from Park et al, 2020. One-way ANOVA, multiple comparison with Bonferroni method; n.s., no significant difference.

 © The Author(s)

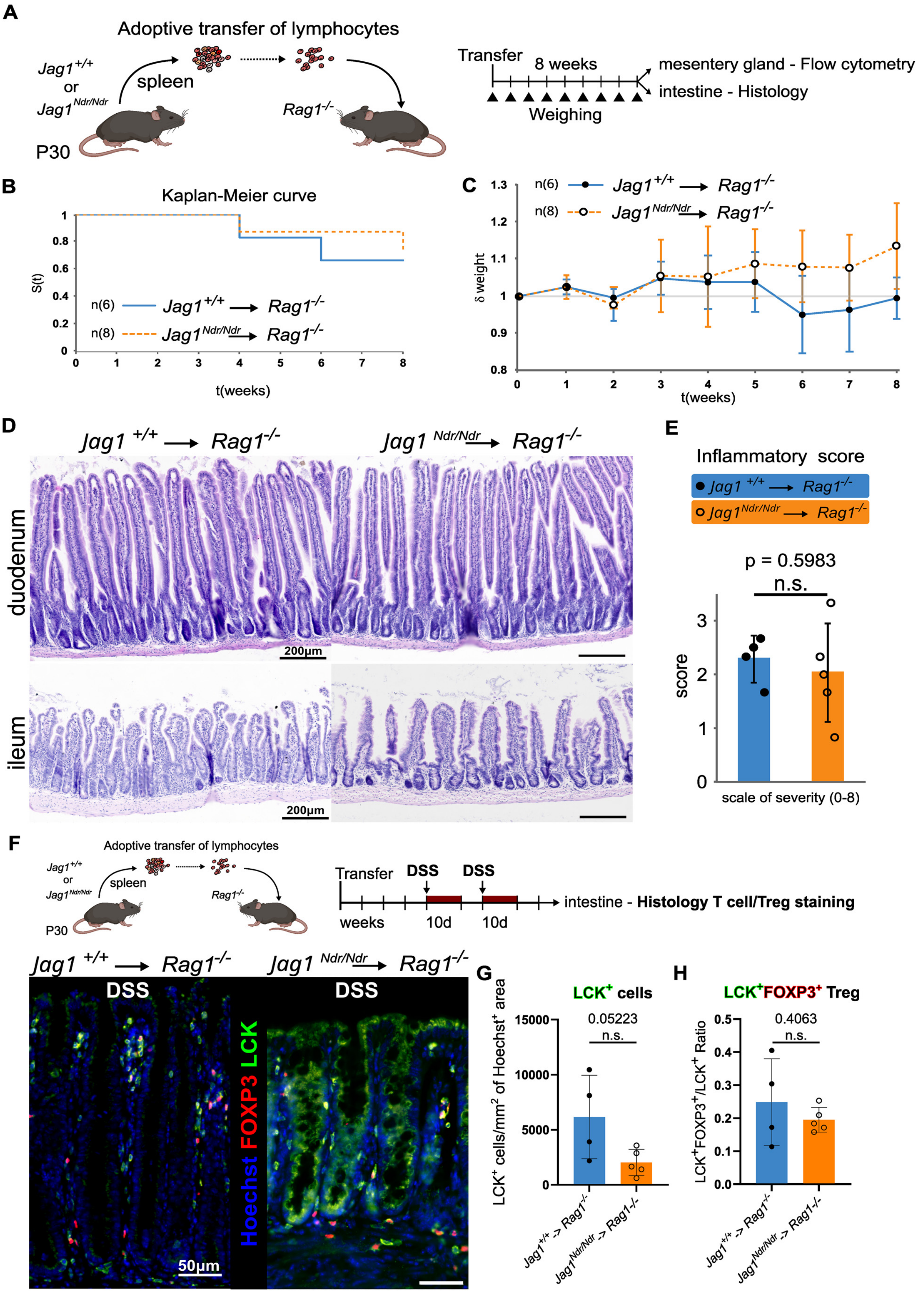
A
Adoptive transfer of lymphocytes
Jag1+/+ or Jag1Ndr/Ndr
spleen
Rag1-/-
P30
Transfer
8 weeks
Weighing
mesentery gland - Flow cytometry
intestine - Histology
B
Kaplan-Meier curve
S(t)
n(6) Jag1+/+ → Rag1-/-
n(8) Jag1Ndr/Ndr → Rag1-/-
t(weeks)
C
δ weight
n(6) Jag1+/+ → Rag1-/-
n(8) Jag1Ndr/Ndr → Rag1-/-
t(weeks)
D
Jag1+/+ → Rag1-/-
Jag1Ndr/Ndr → Rag1-/-
duodenum
ileum
200μm
E
Inflammatory score
Jag1+/+ → Rag1-/-
Jag1Ndr/Ndr → Rag1-/-
p = 0.5983
n.s.
score
scale of severity (0-8)
F
Adoptive transfer of lymphocytes
Jag1+/+ or Jag1Ndr/Ndr
spleen
Rag1-/-
P30
Transfer
DSS
DSS
weeks
10d
10d
intestine - Histology T cell/Treg staining
Jag1+/+ → Rag1-/-
Jag1Ndr/Ndr → Rag1-/-
DSS
DSS
Hoechst FOXP3 LCK
50μm
G
LCK+ cells
0.05223
n.s.
LCK+ cells/mm2 of Hoechst+ area
Jag1+/+ -> Rag1-/-
Jag1Ndr/Ndr -> Rag1-/-
H
LCK+FOXP3+ Treg
0.4063
n.s.
LCK+FOXP3+/LCK+ Ratio
Jag1+/+ -> Rag1-/-
Jag1Ndr/Ndr -> Rag1-/-

Figure EV4. *Jag1*$^{Ndr/Ndr}$ T cells are not autoimmune as shown by the transfer to *Rag1*$^{-/-}$ hosts.

(**A**) Schematic of the experiment. (**B**) Survival curve of the *Rag1*$^{-/-}$ animals over the course of 8 weeks following transfer with *Jag1*$^{+/+}$ ($n = 6$) or *Jag1*$^{Ndr/Ndr}$ ($n = 8$) T cells. (**C**) Relative quantification of *Jag1*$^{+/+}$→*Rag1*$^{-/-}$ and *Jag1*$^{Ndr/Ndr}$→*Rag1*$^{-/-}$ mouse weight normalized to its original value on day 0 after T cell transfer over 8 weeks (1 = 100% of original weight, mean ± SD, n = 6–10 mice). (**D**) Representative H&E staining of intestinal sections (duodenum—top, ileum—bottom) performed 8 weeks after T cell transfer. (**E**) Comparison of the intestinal inflammatory score calculated using the H&E-stained slides of intestinal sections from the *Jag1*$^{+/+}$→*Rag1*$^{-/-}$ ($n = 4$) and *Jag1*$^{Ndr/Ndr}$→*Rag1*$^{-/-}$ ($n = 5$) mice. (**F–H**) Representative immunofluorescent images of sections from *Jag1*$^{+/+}$→*Rag1*$^{-/-}$ (left) and *Jag1*$^{Ndr/Ndr}$→*Rag1*$^{-/-}$ (right) mice intestine after DSS, stained for LCK and FOXP3, counterstained with Hoechst (**F**). Quantification of average LCK^{+} T cell count/Hoechst area (**G**), and LCK^{+}/FOXP3^{+} Treg/LCK^{+} ratio (**H**) Means ± SD, Statistical analysis was performed by unpaired, two-tailed Student's t-test.

© The Author(s)

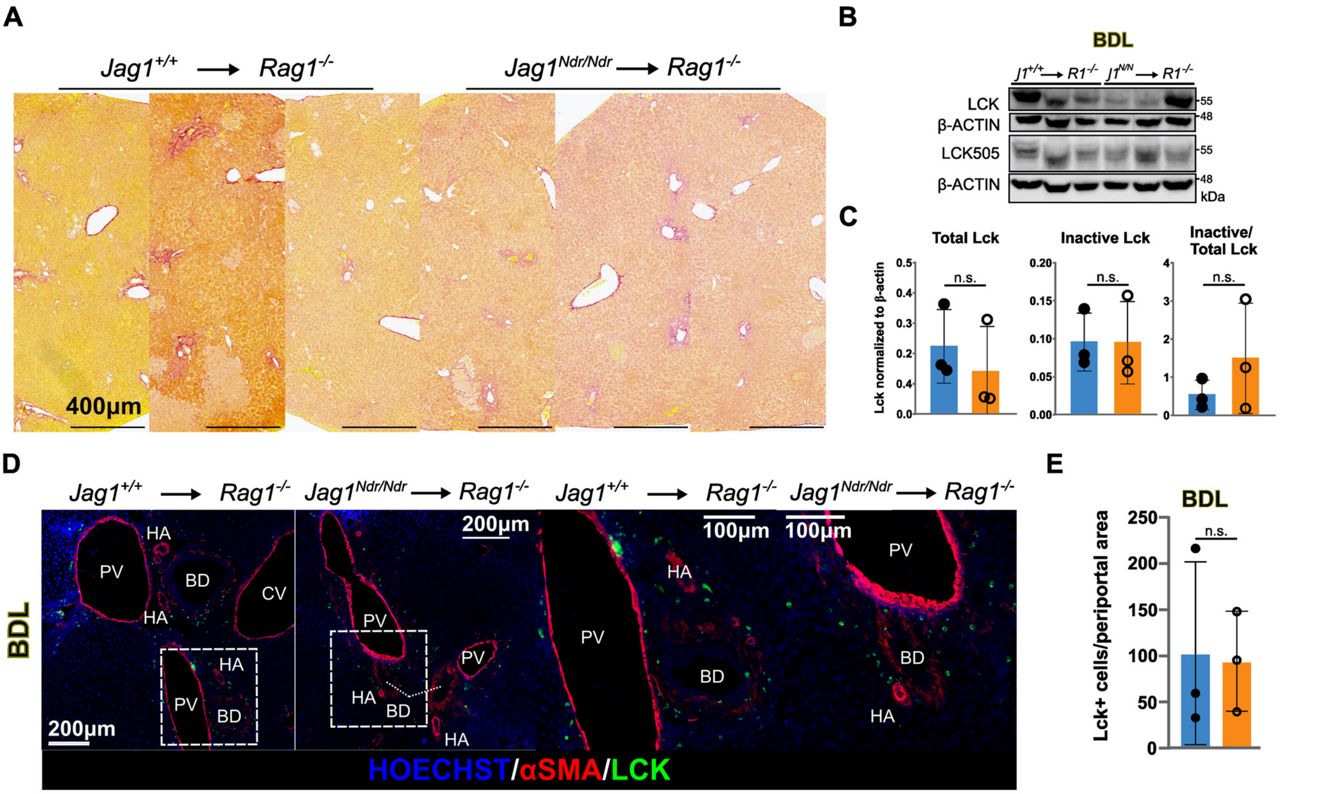

F

	Diagnosis	Manuscript ID	Sex	Age at biopsy	Paucity	Fibrosis	Cholestasis	Manuscript ID Andersson et al. 2018
ctrl2	Autoimmune hepatitis	AIH1	M	14				Control 1M
ctrl3	PFIC2 (BSEP)	PFIC2	F	8				Control 3F
ctrl4	Autoimmune hepatitis	AIH2	F	8				Control 2M
ctrl5	Autoimmune hepatitis with PSC	AIH_SCH	F	4				Control 4F
alg1	Alagille Syndrome	ALGS1	M	3	**	2	****	Alagille 3M
alg2	Alagille Syndrome	ALGS2	F	10	****	1	**	Alagille 5F
alg3	Alagille Syndrome	ALGS3	M	1	**	1	0	Alagille 1M
alg4	Alagille Syndrome	ALGS4	M	18	**	0	***	Alagille 2M
alg5	Alagille Syndrome	ALGS5	F	3	0	0	*	Alagille 4F
https://www.ncbi.nlm.nih.gov/geo/query/acc.cgi								

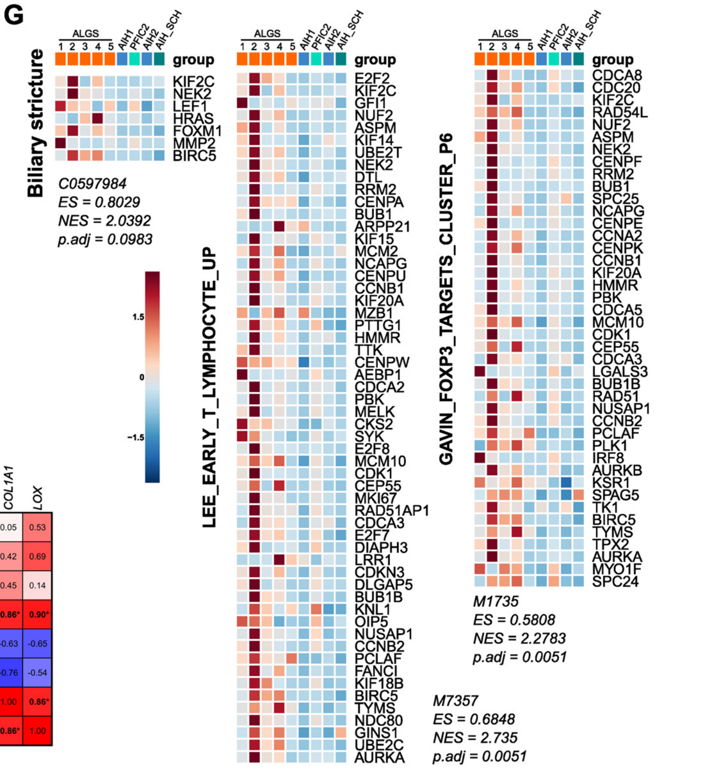

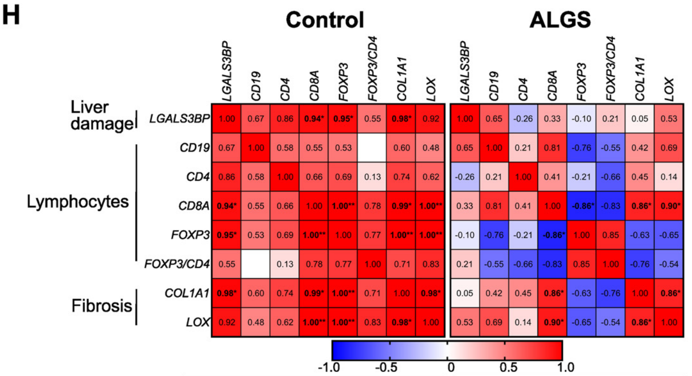

p-values (Control)

	LGALS3BP	CD19	CD4	CD8A	FOXP3	FOXP3/CD4	COL1A1	LOX
LGALS3BP		0.327	0.137	**0.056**	**0.047**	0.454	**0.023**	0.081
CD19	0.327		0.425	0.445	0.474	0.998	0.403	0.524
CD4	0.137	0.425		0.343	0.308	0.865	0.256	0.379
CD8A	**0.056**	0.445	0.343		**0.003**	0.220	**0.008**	**0.004**
FOXP3	**0.047**	0.474	0.308	**0.003**		0.227	**0.005**	**0.005**
FOXP3/CD4	0.454	0.998	0.865	0.220	0.227		0.293	0.171
COL1A1	**0.023**	0.403	0.256	**0.008**	**0.005**	0.293		**0.019**
LOX	0.081	0.524	0.379	**0.004**	**0.005**	0.171	**0.019**	

p-values (ALGS)

	LGALS3BP	CD19	CD4	CD8A	FOXP3	FOXP3/CD4	COL1A1	LOX
LGALS3BP		0.235	0.668	0.589	0.877	0.732	0.935	0.360
CD19	0.235		0.729	0.094	0.139	0.334	0.482	0.198
CD4	0.668	0.729		0.490	0.735	0.230	0.442	0.821
CD8A	0.589	0.094	0.490		**0.060**	0.083	**0.060**	**0.037**
FOXP3	0.877	0.139	0.735	**0.060**		0.067	0.252	0.231
FOXP3/CD4	0.732	0.334	0.230	0.083	0.067		0.140	0.349
COL1A1	0.935	0.482	0.442	**0.060**	0.252	0.140		**0.062**
LOX	0.360	0.198	0.821	**0.037**	0.231	0.349	**0.062**	

© The Author(s)

Figure EV5. T cells, present in the periportal area after bile duct ligation of *Jag1$^{Ndr/Ndr}$* transplanted mice, are enriched in patients with ALGS and anti-correlate with fibrosis.

(**A**) Representative Sirius red staining in Zone 3 of *Jag1$^{+/+}$→Rag1$^{-/-}$* and *Jag1$^{Ndr/Ndr}$→Rag1$^{-/-}$* mice after BDL. (**B**, **C**) Western blot of total LCK, inactive Tyr505 LCK and β-ACTIN levels in LLL lysates from *Jag1$^{+/+}$→Rag1$^{-/-}$* and *Jag1$^{Ndr/Ndr}$→Rag1$^{-/-}$* mice ($n = 3$ each) after BDL (**B**) and respective quantification (**C**). Graph represents mean ± SD. (**D**, **E**) Representative immunofluorescent images of cryosections from the left lateral lobe of *Jag1$^{+/+}$→Rag1$^{-/-}$* (left) and *Jag1$^{Ndr/Ndr}$→Rag1$^{-/-}$* (right) mice after BDL treatment, stained with antibodies against Lck and SMA (**D**), and respective quantification of Lck^{+} in the periportal area (**E**), ($n = 3$ each). Graph represents mean ± SD, Statistical analysis was performed by unpaired, two-tailed Student's t-test. (**F**) List of patient samples from Andersson et al, 2018, re-analyzed in this study. Asterisk indicates severity not significance. (**G**) Heatmap of gene sets over- and under-represented in ALGS and control patients. Significance was confirmed via false-discovery rate (FDR) statistic with 25% cutoff. (**H**) Pearson correlation of liver damage marker *LGALS3BP*, markers of lymphocytes *CD19, CD4, CD8A*, *FOXP3* and markers of fibrosis *COL1A1*, *LOX* mRNA expression in patients with ALGS and control patients (p values *≤0.06; **≤0.005, are indicated below the heatmap). CV central vein, PV portal vein, BD bile duct, HA hepatic artery, AIH autoimmune hepatitis, SCH sclerosing cholangitis, PFIC2 Progressive familial intrahepatic cholestasis type 2, (N)ES (normalized) enrichment score. Fisher Exact test was used for the gene set enrichment analysis; LLL left lateral lobe, CV central vein, PV portal vein, BD bile duct, HA hepatic artery.

 © The Author(s)
